# Acral melanoma detection using dermoscopic images and convolutional neural networks

**DOI:** 10.1186/s42492-021-00091-z

**Published:** 2021-10-07

**Authors:** Qaiser Abbas, Farheen Ramzan, Muhammad Usman Ghani

**Affiliations:** Department of Computer Science, University of Engineering and Technology, 54890 Lahore, Pakistan

**Keywords:** Deep learning, Acral melanoma, Skin cancer detection, Convolutional networks, Dermoscopic images, Medical image analysis, Computer based diagnosis

## Abstract

Acral melanoma (AM) is a rare and lethal type of skin cancer. It can be diagnosed by expert dermatologists, using dermoscopic imaging. It is challenging for dermatologists to diagnose melanoma because of the very minor differences between melanoma and non-melanoma cancers. Most of the research on skin cancer diagnosis is related to the binary classification of lesions into melanoma and non-melanoma. However, to date, limited research has been conducted on the classification of melanoma subtypes. The current study investigated the effectiveness of dermoscopy and deep learning in classifying melanoma subtypes, such as, AM. In this study, we present a novel deep learning model, developed to classify skin cancer. We utilized a dermoscopic image dataset from the Yonsei University Health System South Korea for the classification of skin lesions. Various image processing and data augmentation techniques have been applied to develop a robust automated system for AM detection. Our custom-built model is a seven-layered deep convolutional network that was trained from scratch. Additionally, transfer learning was utilized to compare the performance of our model, where AlexNet and ResNet-18 were modified, fine-tuned, and trained on the same dataset. We achieved improved results from our proposed model with an accuracy of more than 90 % for AM and benign nevus, respectively. Additionally, using the transfer learning approach, we achieved an average accuracy of nearly 97 %, which is comparable to that of state-of-the-art methods. From our analysis and results, we found that our model performed well and was able to effectively classify skin cancer. Our results show that the proposed system can be used by dermatologists in the clinical decision-making process for the early diagnosis of AM.

## Introduction

Skin cancer is an unrestrained, irregular growth of skin cells. This occurs because of unrepaired DNA injury to skin cells or inherited faults [1]. As a result, the top of the skin cells grows rapidly and produce malignant tumors. Skin cancer is categorized into two major types: melanoma and non-melanoma. Melanoma is a type of skin cancer that begins in melanocytes, which are skin pigment control cells. According to recent reports [2], the incidence of skin cancer is increasing, and the number of people who die each year from skin cancer has nearly doubled since 1990. Among other cancer types, melanoma is the deadliest, and is responsible for the death of approximately 55,000 people each year [3]. Melanoma skin cancer is further classified into four subtypes: nodular melanoma, superficial spreading melanoma, acral melanoma (AM), and nodular lentigo melanoma. Acral lentiginous melanoma (ALM) is most prevalent in people with darker skin, such as people of African, Hispanic, and Asian origin [1]. This is not caused by sun exposure. It accounts for less than 5% of skin cancers with melanoma. ALM occurs as a small, flat spot of discolored skin, sometimes dark brown or black. This expands outward through the skin’s surface until it starts expanding deeper into the skin. It normally grows on the soles, palms, or even under the nails. The pathogenesis of ALM remains unknown. It has a worse prognosis than other types of melanomas [4].

Early detection of AM is a difficult task because of the similar visual resemblances between malignant tumors and normal moles, and even the most experienced doctors cannot diagnose accurately [1]. Different medical imaging and biopsy techniques have been applied to diagnose melanoma, such as dermatoscopy, CT scans, and ultrasound. Of these tests, major tests are not required for early-stage melanomas. However, dermatoscopy is usually used to analyze skin lesions for the early diagnosis of skin cancer, especially melanomas and nevus. Experts and trained dermatologists are required to diagnose melanomas at early stages. However, human error is still possible, and an experienced dermatologist may misdiagnose AM, which can be a serious, potentially life-threatening problem [5].

To overcome this, computer-aided diagnosis (CAD) systems have been proposed by different researchers. Image processing and machine learning techniques are widely used in research in which dermoscopic images are analyzed through the CAD system. This helps dermatologists in their early decision-making and prevents misdiagnosis. Dermatologists make use of three clinical diagnosis rules to differentiate melanoma from nevus: the ABCDE rule [6] (Asymmetry, Border, Color, Diameter, Evolve), the 7 point checklist, and the Menzies method [7].

Traditional machine learning techniques require extracted features to classify and diagnose skin cancer. However, this feature extraction process is very difficult and time-consuming [8]. To overcome this feature engineering process, deep learning methods have been developed for lesion analysis and feature extraction. The main benefit of using deep learning methods is that the dermoscopic feature extraction process can be skipped as the algorithms extract dermoscopic features [9]. ALM is more fatal because of late diagnosis and acral presentation [4]. Nevertheless, the development of an automated diagnostic algorithm using dermoscopic features of ALM has been slow because of the rarity of AM. Therefore, there is a lack of sufficient data for automatic diagnosis [4]. Thus, few researchers have investigated the detection of ALMs. For example, earlier studies only focused on categorizing skin cancer into melanoma and nevus [8]. There are only a few studies where researchers have worked on AM detection [4, 10]. The classification of melanoma subtypes is very important for timely diagnosis, and it can increase the patient’s survival rates.

In this research, an automated end-to-end deep learning framework is presented for the feature extraction and classification of AM in dermoscopic images. Various dermoscopic artifacts were removed by image processing. To solve the limited data issues, a data augmentation and transfer learning approach is utilized. Dermoscopic images of AM and acral nevus were used for detection and classification. Three deep learning models were trained using a preprocessed dataset. Our proposed model, a 7-layered deep convolutional neural network (CNN), was designed and trained from scratch. Although transfer learning performs well on a variety of image recognition tasks, it requires more computational resources and time for convergence [11]. Another problem with transfer learning is that when working with a small number of samples, the large deep learning models might lead to overfitting problems that can affect the final classifier performance [9]. Therefore, we first propose a simple CNN model built from scratch. In our second approach, weights were initialized from the pre-trained ResNet-18 and AlexNet models. Transfer learning has shown promising results in various medical imaging classification and segmentation tasks, such as ultrasound classification [12], left ventricle detection [13], skin lesion segmentation [14], and malignant tumor detection [15]. Therefore, we utilized two networks for transfer learning. For this purpose, the last dense layers of the pre-trained networks were replaced with dense layers with two neurons. For ResNet-18, the last four convolutional layers were also trained on our dataset, and for AlexNet, the convolution base was frozen, and only the classification layer was trained. Our experiments showed promising results for classifying AM.

This paper is organized as follows. Introduction and Related work sections present the introduction and literature review, respectively. A detailed description of data augmentation, preprocessing techniques, and CNNs is described in Methods section. The experimental details along with the data and results are reported in Experiments and results section. Discussion section is the discussion, and Conclusions section presents the conclusion.

## Related work

A thorough study of existing methods to detect melanoma was performed, and the proposed methods were formulated to validate the superiority of our approach. In this section, the summarized results of all related research are presented. Various machine-learning techniques have been developed for melanoma diagnosis. Different feature extraction and classification techniques were developed for dermoscopic data such as artificial neural networks (ANNs) and support vector machines (SVMs). Additionally, image processing approaches have been applied pre-processing and artifact removal for dermoscopic image analysis.

In the research proposed by Alquran et al. [6], the dermoscopy image database was preprocessed and segmented using the thresholding technique. The features were extracted using the gray level co-occurrence matrix and ABCD rule [16]. Then, classification was performed using the SVM. The results demonstrated an accuracy of 92.1% for the classification. In another study, conducted by Poornima and Shailaja [17], a method was presented for the detection of melanoma using image processing techniques, such as local binary patterns and grayscale conversion. The skin lesion image was fed as input to the system, after which the image was converted to grayscale, and a local binary pattern method was applied. Dermoscopic features such as color, area, perimeter, and texture were extracted, and segmentation was performed using the active contour method. The active contour method extracts objects with high contrast against the background. The extracted features were used to train an SVM algorithm to classify cancer as melanoma or non-melanoma.

Antony et al. [18] proposed a machine learning technique to classify skin lesions in melanoma and non-melanoma. The image was processed, segmented, and features were extracted. For segmentation purposes, morphological operations such as erosion and dilation were used to segment the lesion from the input image. Subsequently, different features, that is, contrast, correlation, and homogeneity, were extracted. Finally, the features are fed into a classification algorithm. An ANN-based model was employed for classification.

In research conducted by Praveenkumar and Dharmalingam [19], a CAD system for melanoma skin cancer, was proposed using ANN. The features of the segmented images were extracted using a two-dimensional wavelet transform method. These images were used in the ANN for the classification of skin cancers. The system achieved an accuracy of 97%. Murugan et al. [8] presented a machine learning-based system for the classification of skin lesions. The watershed algorithm was applied for the segmentation of the lesion, which increased the precision of the automated system. The performances of three machine learning algorithms were compared: k-nearest neighbor (KNN), SVM, and random forest, were compared. The results showed that SVM performed well on the dataset.

Traditional machine-learning techniques require handcrafted features. However, it is difficult to manually extract features for automatic classification of dermoscopic images. By learning problem-specific features, robust automated systems can be developed. Deep learning algorithms are being utilized in dermoscopy for automated diagnosis. In deep learning methods, automatic feature extraction helps achieve robust and improved classification performance.

In this regard, several researchers have used deep learning. The most recent work in AM diagnosis was performed by Yu et al. [10]. The researchers applied a CNN to a dermoscopic dataset obtained from Severance Hospital. A total of 750 images were obtained from patients, of which nearly half were of AM and the rest of the images were of acral benign nevi. The transfer learning approach was applied to train the visual geometry group 16 (VGG16) pre-trained model. This model achieved 80% accuracy, 92% sensitivity, and 75% specificity.

Hosny et al. [9] employed transfer learning to detect melanomas on the Hospital Pedro Hispano (PH2) dataset. Their experiments showed that the classification performance can be enhanced by eliminating different artifacts in dermoscopic data. The images were preprocessed by removing hairs in the images, and AlexNet was used as a pre-trained CNN for feature extraction. An accuracy of 98.33% was achieved for melanoma detection.

This study performed by Kassani SH and Kassani PH [20], aimed to compare the different deep learning architectures for melanoma detection. The latest deep learning architectures have been utilized to detect melanoma in dermoscopic images. Images were preprocessed to enhance image quality and remove noise from the images. The data augmentation technique was used to reduce overfitting. Their experiments showed that data augmentation and image preprocessing significantly increased classification rates. Their experiments showed 92% accuracy, 93% precision, and 92% recall.

Hosny et al. [15] proposed an automated skin lesion classification system using transfer learning and pretrained CNNs. Three datasets MEDNODE, DERM, and International Skin Imaging Collaboration (ISIC) were used for testing and training in their experiments. Their experiments proved that augmentation and transfer learning can increase the classification rates. Their models achieved an accuracy of 87.31%, 62.02% sensitivity, 79.07% specificity, and 73.07% precision on the ISIC dataset using image augmentation techniques. Data augmentation techniques improved the detection results with 95.91% accuracy, 88.47% sensitivity, 93.00% specificity, and 92.34% precision.

In their experiments, Brinker et al. [21] developed a deep learning system that outperformed 136 out of 157 experienced dermatologists. The results obtained from the system were compared with board-certified dermatologists, where the system outperformed 136 out of 157 in the melanoma detection task. A total of 12,378 dermoscopic images from the ISIC dataset were used to train the deep learning system. Of these images, 100 images were used to compare the performance of the system with that of human experts. For outlier detection, the local outlier factor method was used. The specificity of the deep learning system was 86.5% compared to that of human experts which was only 60%. The sensitivity was 74.1% for both the doctors and deep learning systems.

In this work, Esteva et al. [22] demonstrated the classification of skin lesions using a single CNN with end-to-end training. A deep CNN was trained on 129,450 clinical images, out of which 3374 were dermoscopic images consisting of 2032 different skin diseases. GoogleNet Inception V3 was adopted for classification, which was pre-trained on the ImageNet database. Their experiments utilized a transfer learning approach to increase the accuracy of the system. The whole problem was divided into three classes, where CNN achieved an accuracy of 72.1% compared to the two expert dermatologists who achieved accuracies of 65.56% and 66%, respectively.

Li and Shen [23] presented three deep learning methods for skin lesion analysis. These were lesion segmentation, lesion classification, and lesion dermoscopic feature extraction. The deep learning method, which comprises two fully convolutional residual networks, was utilized to extract the lesion segmentation and classify the lesion. The ISIC 2017 dataset of dermoscopic images was used for training and evaluation of the system. Data augmentation, such as rotation, flipping, and image enhancement, was utilized to increase the accuracy.

Salido and Ruiz [24] proposed a system that preprocesses the images by removing unwanted objects, such as hair, and then segmenting the skin lesion automatically. After removing artifacts and noise from the images, a deep CNN was developed that was applied to both processed and unprocessed images. Their experiments showed that the processed images demonstrated high classification accuracy. Their system achieved 93% accuracy and sensitivity in the 84–94% range.

Sherif et al. [25] experimented with deep CNNs for the detection and classification of melanoma. The ISIC 2018 dataset was used to train the system. The system was tested and validated using the same dermoscopic image dataset. Their system achieved an accuracy of 96.67% for melanoma detection tasks. In this work presented an algorithm that was able to differentiate the furrow and ridge patterns of lesions on the hands and feet for AM detection. Image processing techniques were used to classify patterns in dermoscopic images. The width ratio of the dark and bright patterns in dermoscopic images was utilized and this information was then used to classify the images. Nearly 300 images were used, and their system achieved 99.7% accuracy with a sensitivity and specificity of 100% and 99.1%, respectively. The related work is summarized in Table 1.

**Table 1:** Literature review of Melanoma classification using deep learning

References	Dataset	Methods	Classification	Accuracy
Antony et al. [18]	Self-collected	ANN	Melanoma	86.66%
Alquran et al. [6]	ISIC	SVM	Melanoma	92.1%
Yang et al. [4]	Self-collected	Ridge and furrow pattern	AM	99%
Esteva et al. [22]	Self-collected	Inception V3	Melanoma	72.1%
Yu et al. [10]	Yonsei University	VGG16	AM	80%
Hosny et al. [9]	PH2	AlexNet	Melanoma	98.33%
Praveenkumar and Dharmalingam [19]	Self-collected	ANN	Melanoma	97%
Li et al. [23]	ISIC	CNNs	Melanoma	82%
Salido and Ruiz [24]	PH2	Deep CNN	Melanoma	93%
Hosny et al. [15]	ISIC, DERM	AlexNet and augmentation	Melanoma	87%
Sherif et al. [25]	ISIC	Deep CNN	Melanoma	96.57%
Murugan et al. [8]	ISIC	SVM, kNN	Melanoma	89.43%, 76.87%
Kassani SH and Kassani PH [20]	ISIC	Deep CNNs	Melanoma	92%
Brinker et al. [21]	ISIC	CNNs	Melanoma	Specificity 86.5%, sensitivity 74.1%

Most of the work in the literature is directed toward the binary classification of melanoma and non-melanoma. Other researchers have worked on non-melanoma skin cancer classifications. In this paper, a fully automated end-to-end deep learning model for an AM detection task is presented using a dermoscopic image dataset. Data augmentation and transfer learning are applied to enhance the classification performance of the system. This approach can be utilized to help physicians in their decision-making process during the early detection of AM.

## Methods

**Fig. 1:**
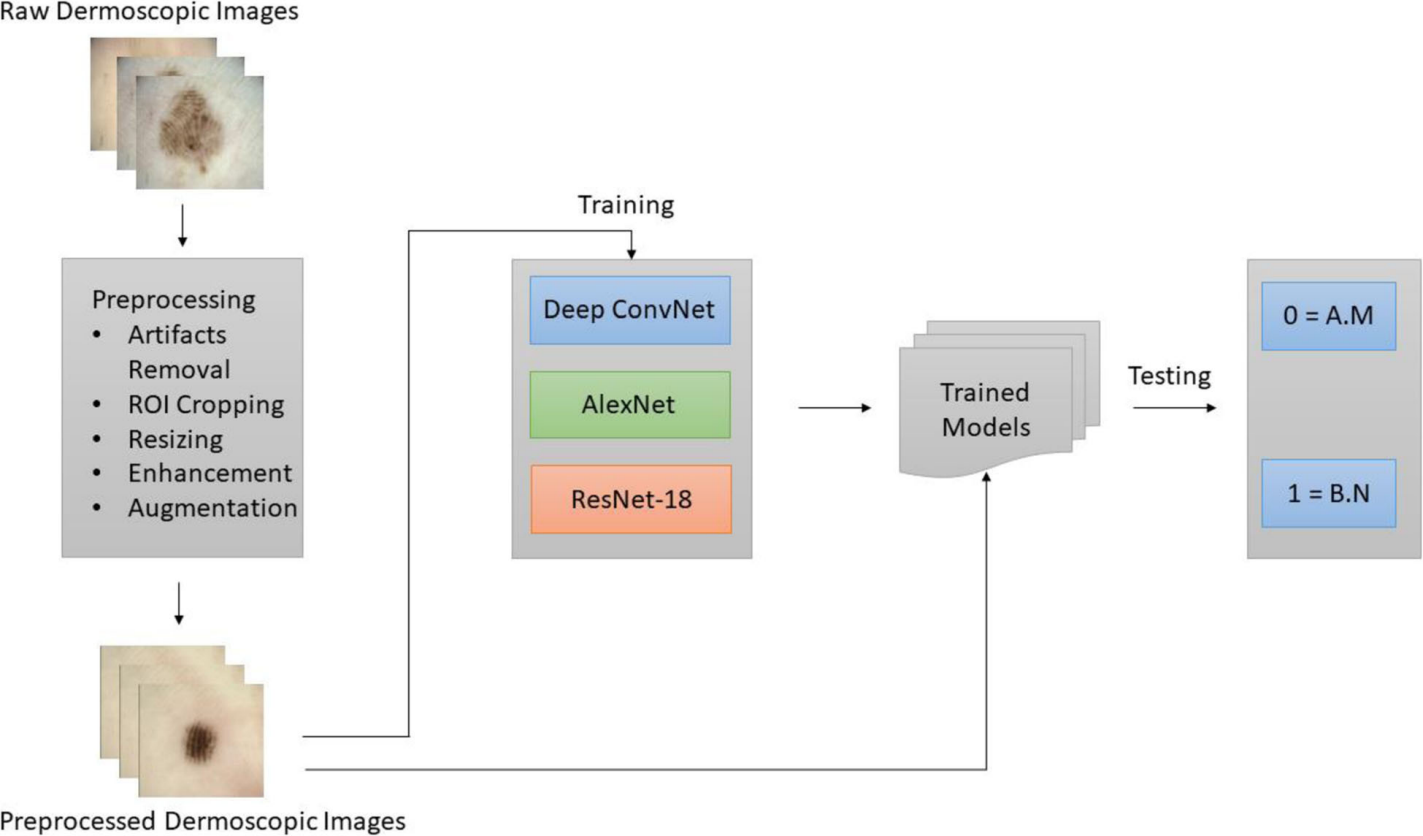
Steps of the proposed methodology

The research methodology includes three stages: pre-processing and data augmentation, second feature extraction, and third classification and prediction. Dermoscopic image data were acquired from a well-known South Korean university hospital [10]. Various preprocessing methods have been applied to remove the dermoscopic artifacts. A preprocessed dataset was used to train the deep learning models. A flowchart of the methodology is shown in Fig. 1.

### Dataset preparation

In this study, 724 dermoscopic images were collected by Severance Hospital in the Yonsei University Health System, Seoul, South Korea. Of these 724 images, 350 were from AM patients and 374 were benign nevus (BN) patients. All diagnoses were confirmed histopathologically. The dataset of images was divided into training, validation, and testing, where 70% of the images were used to train the algorithm and 20% were used for validation. The remaining 10% were utilized to evaluate the performance of the algorithm on unseen images.

### Dataset preprocessing

The raw dermoscopic images were high-resolution images, which were computationally expensive. In addition, these dermoscopic artifacts are challenging for automated classification. To overcome this issue, these challenges were solved using the following preprocessing techniques.

### Artifacts removal

The raw dermoscopic images contain several artifacts that may lead to poor performance of the deep learning model. Some of the artifacts found in dermoscopic images are dark corner artifact frames, ruler marking, hairs, etc. These were removed using cropping and image processing techniques, as described by ref. [26].

### ROI cropping and resizing

**Fig. 2:**
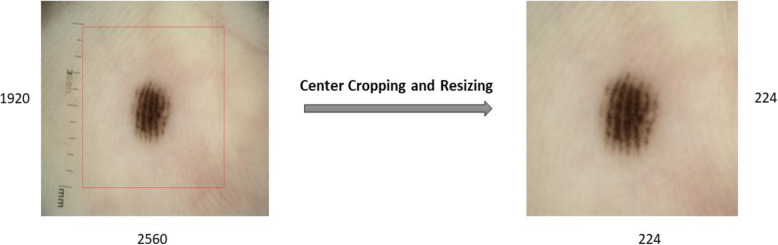
Preprocessing for skin lesion center cropping and resizing

The original dataset contains 724 skin lesion 2D RGB images with high resolution (2560 × 1920). These high-resolution images of some lesions require a high computational cost. To fit the input size of the CNN, the images must be resized. As directly resizing images may lead to shape distortion of skin lesions, the first ROI of the skin lesion was as practiced by the authors in ref. [23] and then resized to low resolution to preserve the features and shape of the skin lesion. The center size was set to 0.7 of the height of the original image, and the image was automatically cropped from the center. As illustrated in Fig. 2, this approach enlarges the lesion area for extracting features and preserves the original skin lesion shape.

### Data augmentation

High-performance deep networks require large datasets. However, in the medical domain, owing to privacy concerns, obtaining large amounts of data is a significant challenge [22]. The original dataset contains only 724 images belonging to two classes of AM and BN, which are not sufficient for a high-performance deep learning model. To overcome this issue, different image augmentation techniques have been applied, as described by Perez et al. [27] in their work. The images were rotated to different angles and flipped to artificially increase the number of images. Rotation angles of 90°, 180°, and 270° were applied to generate new images. In addition to these image transformations, flipping the image upside down and from left to right was also applied to increase the dataset size. Six samples were generated from a single training sample using the augmentation method. After applying different transformations, a six-fold increase in data was generated. Table 2 summarizes the details of the original dataset and the preprocessed augmented dataset.

**Table 2:** Dataset details

Dataset	Total images	AM	BN
Yonsei University [10]	724	350	374
Augmented (ours)	4344	2100	2244

### Deep learning models

In this section our deep learning network, which was employed for feature extraction and classification tasks, is described.

#### Model 1 deep CNN

The proposed model is a 7-layer deep convolution neural network. The input to the deep ConvNet model is RGB images of size 224 × 224 × 3. This deep ConvNet model consists of five convolutions and two fully connected layers. MaxPooling was applied after each convolutional layer. The outputs from the convolution layers are normalized using batch normalization layers. The nonlinear activation function ReLU was adopted as the activation function. The first layer applied eight convolutional filters with a size of 3 × 3. After this convolution operation, batch normalization was applied to normalize the input. This sometimes serves as a regularization and increases the network learning speed. ReLU is applied after this, followed by 2 × 2 MaxPooling. This block was repeated four times. However, the number of convolutional filters was increased to 16, 32, 64, and 128 in successive layers. After the final network block, the output from the last convolutional block is flattened to form a 256 neuron fully connected layer, followed by a 2 neuron fully connected layer. In the final layer, the SoftMax layer was applied to classify the inputs into the predicted labels. Dropout was applied to reduce overfitting, with a dropout value of 0.3. Data augmentation was also applied to handle the overfitting in the network. Our network contained 919,346 parameters. SGD was used as an optimization algorithm, and categorical cross entropy was used as the loss function. The cross-entropy loss is defined as

$$\begin{array}{*{20}c}CE=-{\sum }_{i}^{c}{T}_{i}\text{log}\left({S}_{i}\right) \left(1\right)\end{array}$$

where T_*i*_ and S_*i*_ are the ground truth and predicted labels for each class *i* in C.

In our case, we have two classes, that is, C = 2 (AM and BN), so the cross-entropy loss can be described as follows:

$$\begin{array}{*{20}c}CE=-{\sum }_{i}^{c=2}{T}_{i}\text{log}\left({S}_{i}\right)= - {t}_{1}\text{log}\left({s}_{1}\right)-\left(1-{t}_{1}\right)\text{log}\left(1-{s}_{1}\right) \left(2\right)\end{array}$$

Where *t*_1_ = ground truth, *s*_1_ = model prediction.

**Fig. 3:**
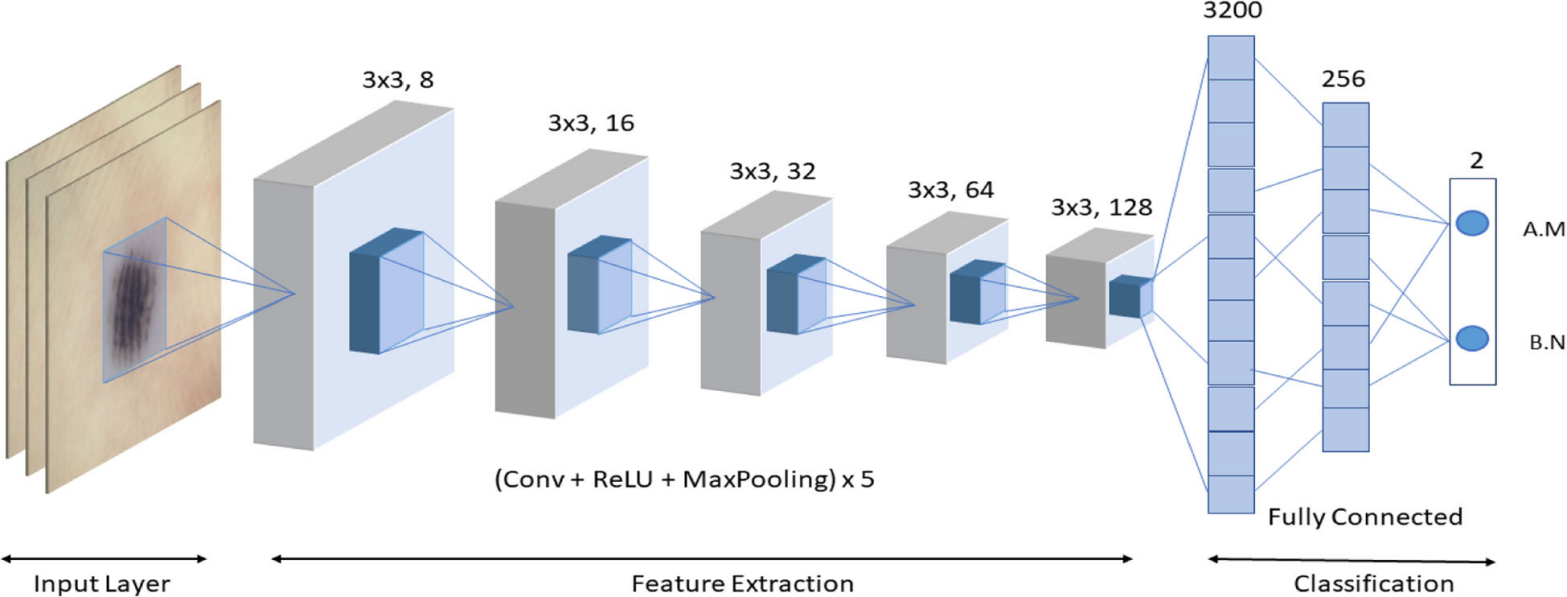
Designed 7-layer deep ConvNet

Figure 3 presents the convolutional network architecture utilized in this study.

#### Model 2 transfer learning with AlexNet

AlexNet is a CNN proposed by Krizhevsky et al. [28]. AlexNet used this network and competed in the ImageNet Large Scale Visual Recognition Challenge in 2012. The network was able to achieve a top-5 error of 15.3%, which was 10.8% lower than the network in the second place. The results of the network showed that the network depth was important for better performance in image classification. Although this was expensive, the use of GPUs made it feasible.

The network architecture contains a total of eight layers, five convolutional layers and three fully connected layers, followed by a SoftMax layer for classification. This architecture uses some special features that help improve the performance of deep ConvNets. It applied max-pooling after convolutional layers and utilized dropout regularization. Additionally, to further train the deep network effectively, the nonlinear rectified linear unit was used as an activation instead of the tanh activation function. This architecture has a total of 60 million parameters.

**Fig. 4:**
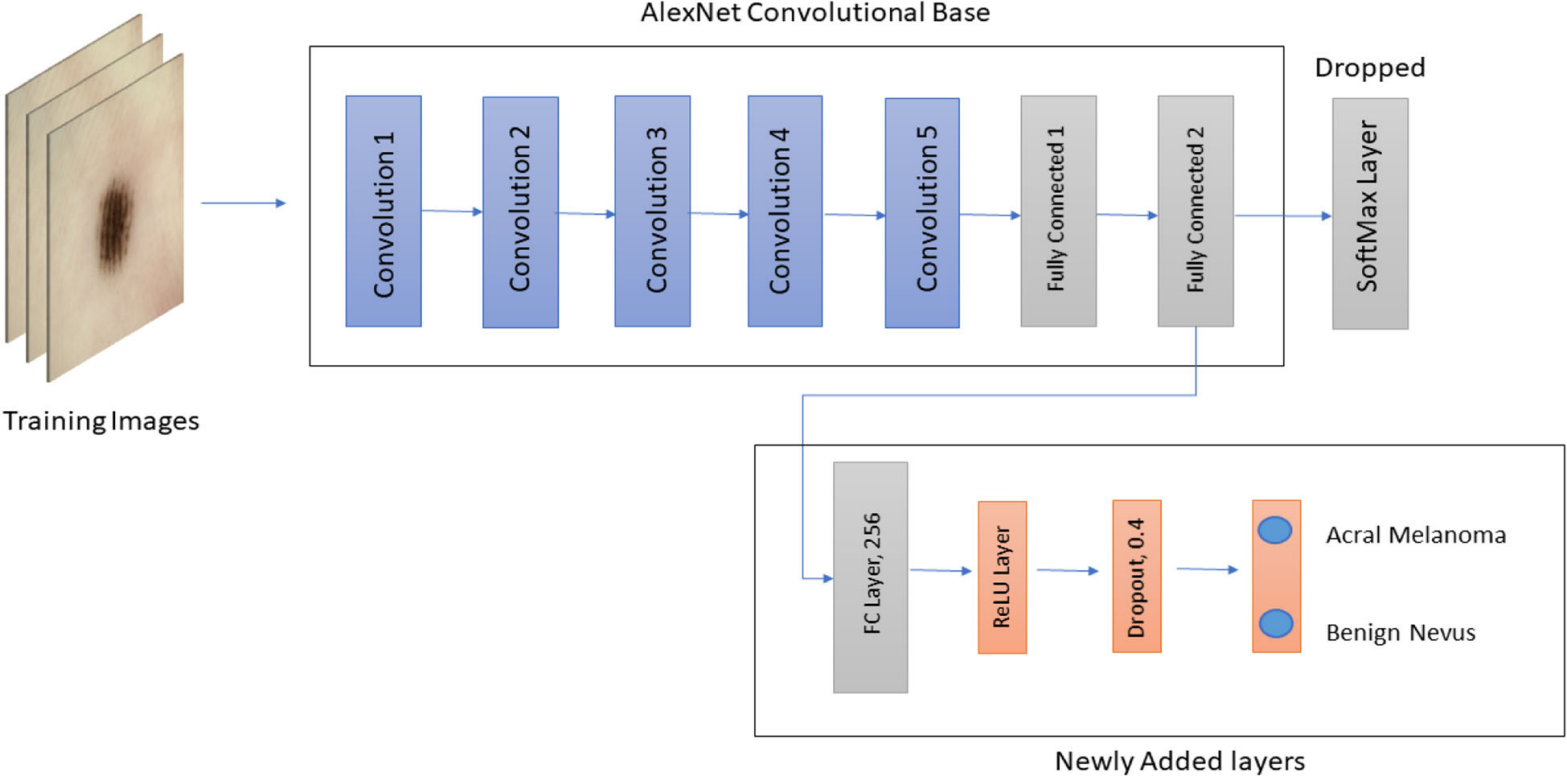
Modified AlexNet model

A pre-trained AlexNet model was utilized, which was originally trained on the ImageNet database. Transfer learning was utilized by replacing the last dense layer of the original network, which consisted of 1000 neurons with 2 neurons. A new fully connected layer with 256 neurons was added, followed by a ReLU activation layer. To overcome overfitting, a dropout layer was added with a value of 0.4. All convolutional layers were used to extract features from dermoscopic images, and the final classifier was trained on our dataset. The configuration of the adopted architecture is shown in Fig. 4.

#### Model 3 finetuning deep residual neural networks

ResNet was proposed by He et al. [29]. A residual learning method is presented to train deeper networks. Deeper networks are difficult to train because of the vanishing gradient and exploding gradient problems; therefore, this residual method was proposed to train deep networks. According to their research, residual learning-based deeper networks achieve better optimization and high accuracy because of the depth of the network. When deep networks start converging, the accuracy becomes saturated. This problem was solved by introducing a residual function. The original network was trained using ImageNet database. ResNet took first place in the 2015 ILSVRC competition with a top 5 error rate of 3.57%.

Network layers are stacked in plain neural networks to learn the anticipated mapping directly. However, in residual networks, residual mapping is learned by the stacking layers. The mapping function, referred to as *H*(*x*), is equipped with a stacked layer, where *x* is the input. The mapping is given by the following equation:

$$\begin{array}{*{20}c}H\left(x\right)=F\left(x\right)+x \left(3\right)\end{array}$$

And the residual mapping function is given by:

$$\begin{array}{*{20}c}F\left(x\right)=H\left(x\right)-x \left(4\right)\end{array}$$

Instead of learning the *H* (*x*) function, the stacked layers learn the residual function *F* (*x*) explicitly. The original mapping function is determined after approximating the residual function as *H*(*x*) = *F*(*x*) + *x*. This mapping function *F*(*x*) + *x* is realized in a feedforward neural network as a residual shortcut connection and performs element-wise addition.

**Fig. 5:**
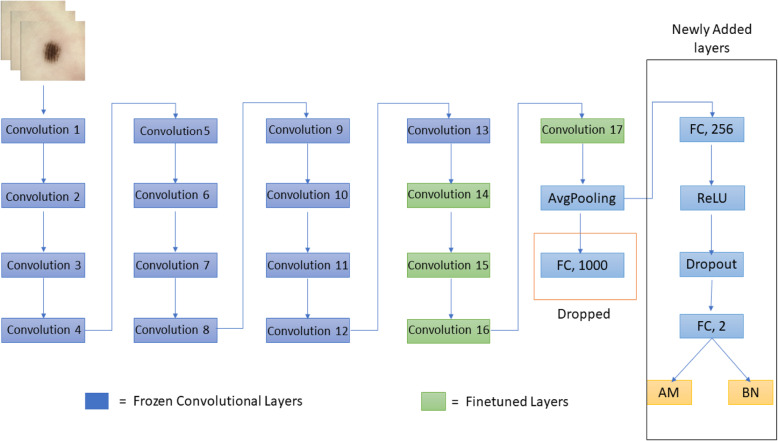
Modified ResNet used for this study

A pre-trained ResNet-18 model was utilized, which was trained on the ImageNet database. The network is illustrated in Fig. 5. The network contains 18 layers, 17 convolutional layers, and a fully connected layer. Transfer learning was performed by replacing the last dense layer of the network, which consisted of 1000 neurons with 2 neurons. All the convolutional layers were frozen, except for the last four layers. The last four convolutional layers and the last fully connected layer were trained on our dataset. Dropout regularization was also applied to prevent overfitting. SGD with momentum was used as an optimizer, and cross-entropy loss was utilized to train the network. Figure 5 shows the configuration of the modified ResNet architecture.

## Experiments and results

This study aimed to classify melanoma subtypes using dermoscopic images. Dermoscopic images of AM and BN were acquired from Yonsei University Hospital. Various preprocessing and augmentation techniques were applied to the dataset. In this section, details of the experiments and results of the deep learning models are discussed.

To train deep learning networks, a cloud GPU service by Google, known as Google Colaboratory, was utilized. Google Colab [30] is a free cloud-based Jupyter notebook environment that allows training of large and complex deep learning algorithms on free GPUs and TPUs. It provides a single 12GB NVIDIA Tesla K80 GPU with 25GBs of RAM. Keras and PyTorch were used as deep learning frameworks. Python was used as the programming language. The details of the experiments are discussed in the next section.

### Experiments with proposed ConvNet model

The preprocessed dermoscopic images were used to train the deep convolutional network model to classify melanoma subtypes. The images were resized to 224 × 224, where each image was a 2D image with three channels, that is, RGB. From these preprocessed images, 70% were used for training and 20% for validation, and 10% was used for testing the unseen test data. The initial learning rate was set to 0.0001, and the weight decay factor was reduced by dividing the learning rate by the number of epochs after every epoch. Stochastic gradient descent with momentum was utilized as an optimizer, where the momentum was set to 0.9. The network was trained for up to 100 epochs. A batch size of 16 was used during training. An average classification accuracy of 91.03% was achieved using a 7 Layer deep convolutional network.

### Experiments using transfer learning

Dermoscopic images were used to fine-tune the deep ResNet model to classify melanoma subtypes. The images were resized to 224 × 224 to match the input size for ResNet. From these images, 70 % were used for training, 20% for validation, and the remaining 10% were used for testing on unseen data. Other parameters were kept the same except for the batch size, which was set to 32 and the learning rate, which was set to 0.001 in the case of ResNet-18. An average classification accuracy of 97.47% was achieved using the pre-trained modified ResNet-18.

In another experiment, AlexNet, which was originally trained on the ImageNet database [31], was fine-tuned. This original network was also trained to classify 1000 categories of images from ImageNet. The same experimental setup was used to train the modified models. An average classification accuracy of 95.87% was achieved using a pre-trained AlexNet model. Table 3 summarizes the testing accuracies of the three deep-learning models for AM classification. The best average accuracy was achieved with the modified pre-trained ResNet-18 model. This improvement is comparable to that of the pre-trained AlexNet model.

**Table 3:** Comparison of deep learning models

Class	Proposed ConvNet	ResNet-18	AlexNet
AM	0.9003	0.9691	0.9619
BN	0.9203	0.9772	0.9555
Accuracy (mean ± std )	0.9103 ± 0.01	0.9747 ± 0.004	0.9587 ± 0.003

### Comparative analysis of deep learning models

**Fig. 6:**
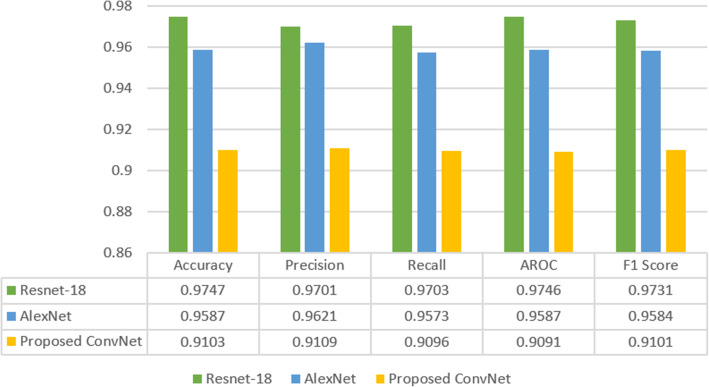
Comparison of deep learning models

In this section, the results obtained from all four deep learning models are compared. Transfer learning delivers state-of-the-art results in image classification as the models are already trained on a large ImageNet database; thus, the model has learned useful low-level features that can be transferred to train these models on new datasets. For medical image analysis, large publicly available datasets are limited to specific domains. As AM is a rare skin cancer that affects the human acral skin areas, a large dataset is not available to train automated systems [4]. In addition, our specific task in this research was to develop a ConvNet for AM classification, and due to rare and infrequent occurrences of this subtype of cancer, a large public dataset is not available. However, data augmentation was applied to enlarge the training dataset and develop an automated classifier. Although our custom-built ConvNet was trained from scratch, this model obtained promising results without using transfer learning. By utilizing effective hyperparameters, we produced classification results that were comparable to the transfer learning approach. Figure 6 summarizes the outcomes of all three model results. Evaluation metrics such as precision, recall, accuracy, F1-Score, and AROC were used to evaluate the models.

**Fig. 7:**
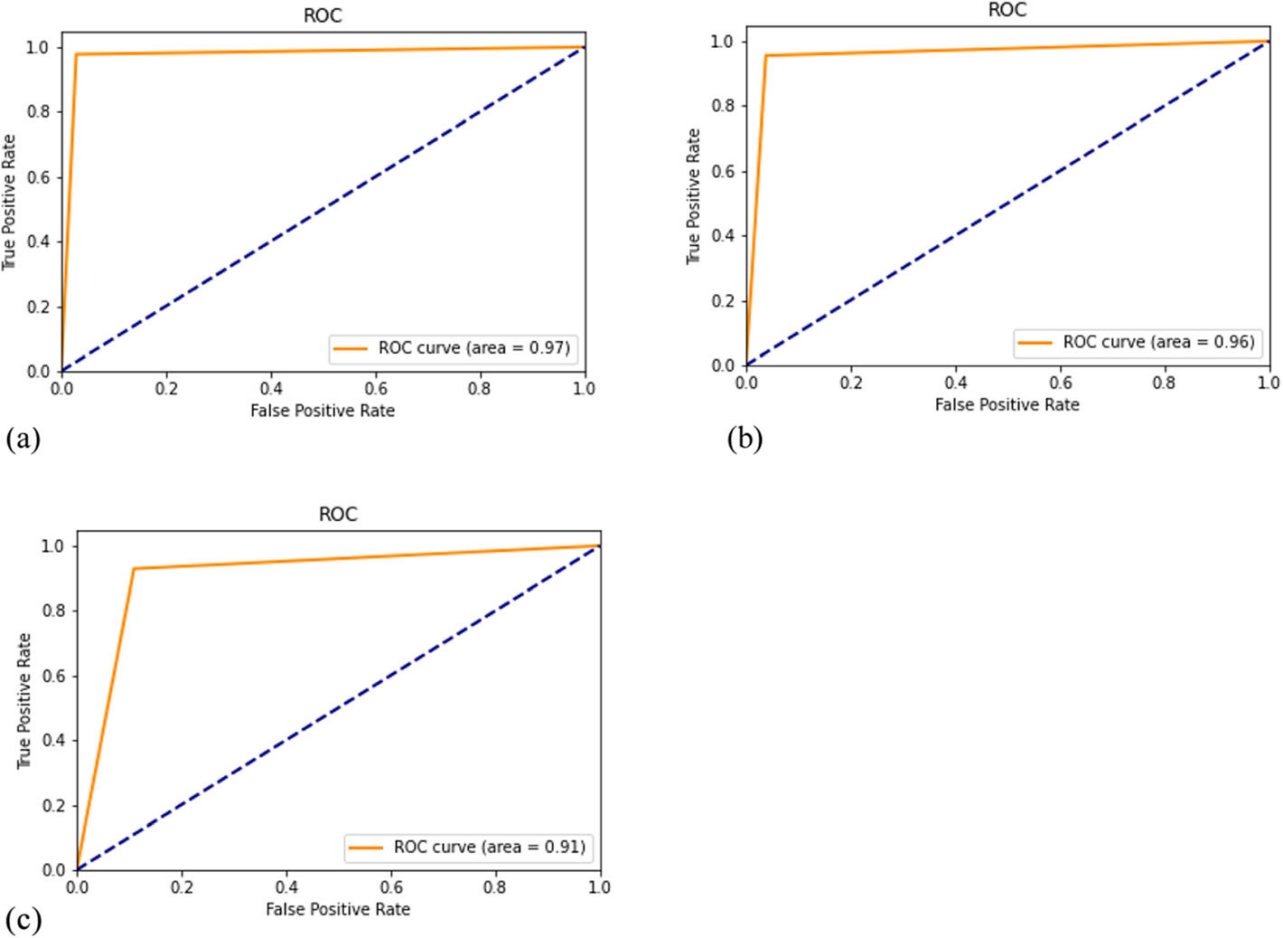
ROC curve. (**a**): ROC curve for modified ResNet model; (**b**): ROC curve for AlexNet model; (**c**): ROC curve for custom deep learning model.

The Fig. 7 present the ROC curves of all three models.

The best value for the ROC curve was obtained by the modified ResNet model [Fig. 7(a)], which was 0.1% more than that of the AlexNet model [Fig. 7(b)] and 0.6% more than that of our proposed Deep ConvNet model [Fig. 7(c)]. Transfer learning has helped increase true positives and true negatives. When we move from ResNet to ConvNet, the decrease in performance is visible, which is justified as transfer learning helps to achieve better performance, resulting in a deep learning paradigm.

## Discussion

Various automated diagnostic methods have been proposed for the early diagnosis of melanoma. However, these methods cannot be applied to AM [4, 5, 10] due to the infrequent occurrence of melanoma in Asians. Automated diagnosis techniques require large datasets that are not available because of the low occurrence rate. To solve the insufficient dataset problem, data augmentation and transfer learning techniques were adopted. Data augmentation techniques such as rotation, translation, and flipping assist in the development of a robust CNN [27]. Transfer learning also helps in the development of a robust classifier using insufficient data, as the features learned by a network can be transferred to the new system. Hence, these methods are required to develop a fully automated system for melanoma detection using small datasets.

Our results showed that the accuracy of our proposed CNN was higher than 90% for all three models. The CNN models showed AUC values greater than 0.9, indicating good discrimination. High sensitivity is essential for effective screening of AM, and our system produces high sensitivity, which is higher than that of a human expert [10]. Therefore, CNNs can be used for the early diagnosis of AM by doctors unfamiliar with the dermoscopy technique.

There are several rule-based automated classification methods, such as the ABCD rule [16] and 7-point scale [7]. This approach requires specific features such as color, size, shape, and lesion boundary. However, these approaches cannot be directly applied to AM due to different features, such as ridge or furrow patterns. Although there is a state-of-the-art automated classification method [4] that shows superior results than our approach, this method cannot be generalized and performs well only for specific lesion patterns such as ridge-and-furrow patterns. Other feature-based automated classification methods cannot be directly applied to AM due to various dermoscopic features such as ridge patterns and furrow patterns. Automated classification approaches using specific features can achieve superior performance [8]. However, it is difficult for an automated learning algorithm to understand the observations of experts. In contrast, deep learning networks do not require extracted features. The system automatically extracts the most interrelated features through learning. Hence, the accuracy of deep learning models is superior to that of traditional machine learning and feature-based models [15].

Recently, various non-invasive devices, such as confocal and photon microscopy, have been presented for the early diagnosis of melanoma [32]. However, these techniques require considerable effort and time to become sophisticated. In contrast several machine learning based methods dermoscopic images border irregularity detection [33, 34], have been applied to but AM depend on many other dermoscopic features. A fully automated diagnosis system using deep learning has the potential to overcome the difficulties faced while learning these techniques. Table 4 summarizes the comparative analysis of automated methods for AM classification.

**Table 4:** Comparative analysis of classification accuracy

Study	Average accuracy	AUC	Recall	Precision
Yu et al. [10]	80%	0.84	92.57%	77.14%
Yang et al. [4]	99.7%	-	100%	99.1%
Deep ConvNet	91%	0.90	90.96%	91.09%
AlexNet	95%	0.95	95.73%	96.21%
Deep ResNet	97%	0.97	97.01%	97.10%

## Conclusions

Melanoma is caused by the abnormal growth of skin cells, which affects the majority of the population worldwide. It has four subtypes that can be fatal if not treated early. Once it increases, malignant tumors can affect nearby healthy cells, resulting in metastatic melanoma disease where the survival rate of patients decreases sharply. Due to the visual similarity between benign and malignant tumors on the skin, it is difficult to diagnose the disease in the early stages. This visual similarity increases the chances of misdiagnosis, which leads to patient death in the long run. The traditional approaches to automatically diagnosing melanoma are limited to binary class classification, in which melanoma is differentiated from a non-melanoma skin lesion. However, melanoma has four further subtypes, such as AM, that have a poor diagnosis.

An end-to-end deep learning model for feature extraction and melanoma subtype classification is presented in this research. Dermoscopic images of AM were acquired from the Yonsei University Acral Melanoma dataset to distinguish AM from acral nevus. Different image preprocessing techniques have been applied for image enhancement and to prepare images for deep learning models. The preprocessed images were fed into the proposed deep learning model for classification. Our custom-built deep ConvNet, which was trained from scratch, achieved 91% accuracy, while the use of transfer learning produced slightly higher accuracies. In comparison to a recent study [10], our modified ResNet-18 model achieved a higher average accuracy. The results from our study indicate that dermoscopy, along with deep learning, can be helpful for the early diagnosis of rare skin cancers such as AM. The same methods can also be applied to other subtypes of melanoma, and this approach is promising for diagnosing skin cancer using dermoscopic images.

## Data Availability

All relevant data that was used in this paper are available publicly. The data can be accessed from figshare at https://figshare.com/s/a8c22c09f999f60a81bd.

## Abbreviations

ALM: Acral lentiginous melanoma
AM: Acral melanoma
BN: Benign nevus
CAD: Computer-aided diagnosis
CNN: Convolutional neural network
KNN: k-nearest neighbor
ANN: Artificial neural network
SVM: Support vector machine
ISIC: International Skin Imaging Collaboration
VGG: Visual geometry group
PH2: Hospital Pedro Hispano
